# Impact of Pneumococcal Conjugate Universal Routine Vaccination on Pneumococcal Disease in Italian Children

**DOI:** 10.1155/2015/206757

**Published:** 2015-08-16

**Authors:** Francesca Fortunato, Domenico Martinelli, Maria Giovanna Cappelli, Vanessa Cozza, Rosa Prato

**Affiliations:** Department of Medical and Surgical Sciences, University of Foggia, 71100 Foggia, Italy

## Abstract

In Italy, the effectiveness of pneumococcal universal vaccination in preventing vaccine-type invasive pneumococcal disease (IPD) in the PCV7/PCV13 shifting period was estimated to be 84.3% (95% CI: 84.0–84.6%) in children <5 years. This study aims at corroborating the estimation of both the effectiveness (VE) of PCVs and its impact in reducing pneumococcal diseases. A 1 : 3 matched-case-control study was conducted among children <5 years old hospitalized for IPD or pneumococcal pneumonia (PP) between 2006 and 2012 in the Puglia region. Moreover, hospitalizations for pneumococcal outcomes in the pre- and postvaccination period and the hospitalization risk ratios (HRRs) with 95% CIs were computed in Italy and in the first eight regions that introduced PCVs in 2006. The overall effectiveness of PCVs was 75% (95% CI: 61%–84%); it was 69% (95% CI: 30%–88%) against IPD and 77% (95% CI: 61%–87%) against PP. PCVs showed a significant impact on IPD and acute otitis media either at a national level or in those regions with a longer vaccination history, with a nearly 40% reduction of hospitalizations for both outcomes. Our findings provide further evidence of the effectiveness of PCVs against pneumococcal diseases and its impact on nasopharyngeal carriage in children <5 years, indicating the importance of maintaining high immunization coverage.

## 1. Introduction

Pneumococcal infection is a major cause of morbidity and mortality worldwide [1]. Among young children and the elderly population,* Streptococcus pneumoniae* causes invasive disease (IPD), such as severe blood infection, meningitis, and pneumonia [2].

Since the introduction of the 7-valent conjugate vaccine (PCV7, containing serotypes 4, 6B, 9V, 14, 18C, 19F, and 23F) in 2000, there has been an overall reduction in the incidence of all-serotype IPD, ranging from 80% in the USA to 30–40% in Europe, in children under 5, despite the reported increase in the incidence of pneumococcal diseases due to non-PCV7 serotypes (NVT) in all age groups [3, 4].

Following the globally observed changes in the overall serotype distribution of* S. pneumoniae*, particularly a rise in serotype 19A, higher-valent pneumococcal conjugate vaccines (PCV10, comprising the additional serotypes 1, 5, and 7F, and PCV13, comprising the additional serotypes 1, 3, 5, 6A, 7F, and 19A) were developed and widely employed to provide improved serotype coverage against pneumococcal diseases. Second-generation PCVs, routinely implemented since 2010, have showed an early impact and effectiveness for reducing pneumococcal infections in both vaccinated and unvaccinated children and in reducing nasopharyngeal carriage of vaccine-serotypes [5–7]. In the first study from Miller et al. in England and Wales, PCV13 effectiveness in preventing IPD was estimated to be 78% (95% CI: −18% to 96%) and 73% (95% CI: 29%–90%) for two priming doses and for one dose over a year, respectively [8]. Moreover, PCV13 showed an early impact on overall pneumococcal nasopharyngeal carriage in young children <2 years with acute otitis media (AOM) [9].

In Italy, PCV7 was available on the market from 2002 to 2010. Between 2006 and 2010, all 21 regions had recommended or introduced the vaccine in their childhood immunization schedule [10, 11]. In May 2010, the Ministry of Health issued a recommendation to replace PCV7 with PCV13 [12]. Finally, the National Vaccination Plan 2012–2014 included the active offer of 3 PCV13 doses in the list of “Essential Health Interventions” for newborns at 3, 5-6, and 11–13 months of age. The plan set the objective to achieve and maintain PCV13 vaccination coverage (VC) of at least 95% in children <24 months of age [13]. Throughout the transition period between PCV7 and PCV13, children who had received one or two doses of PCV7 completed their immunization series with PCV13. One PCV13 catch-up dose was recommended for children fully vaccinated with PCV7 [12, 14].

In 2013, a survey conducted among 14 Italian regions showed that PCV vaccination coverage in children aged 24 months progressively increased from 2005 to 2009 birth cohort, with a considerable variability between regions, from 44.7% to 98.5% in 2011. Due to the reduction in circulation of the vaccine-serotypes, the incidence of IPD in children aged 0–4 years decreased from 7.1 per 100,000 in 2008 to 3.8 per 100,000 in 2012 [10].

The Puglia region (southeast Italy, ~4,000,000 habitants) was one of the first eight Italian regions to introduce PCVs in the childhood immunization schedule in 2006 [15, 16]. Vaccination coverage in children under 24 months of age increased from 75.3% in the 2006 birth cohort (PCV7 only) to 95.1% in the 2010 birth cohort (PCV7/PCV13) [17] and was 93% in the 2011 birth cohort (PCV13 only). In our previous study (Martinelli et al.), the overall effectiveness of the pneumococcal immunization programme in preventing vaccine-type IPD during and after the PCV7/PCV13 shifting in the immunization schedule was estimated at 84.3% (95% CI: 84.0%–84.6%). The impact of vaccination on hospitalizations for pneumococcal diseases in children under 5 years of age recorded the most important reduction for pneumococcal pneumonia (HRR: 0.43, 95% CI: 0.21–0.90), followed by IPD (HRR: 0.72, 95% CI: 0.21–2.43), acute otitis media (HRR: 0.75, 95% CI: 0.65–0.88), and all-cause pneumonia (HRR: 0.92, 95% CI: 0.86–0.99) [17].

This study aims at corroborating the estimation of both the effectiveness of PCVs and its impact in reducing severe pneumococcal diseases in children under 5 years of age in Italy.

## 2. Methods

### 2.1. PCVs Programme Effectiveness

To estimate the vaccination programme effectiveness (VE) in the PCV7/PCV13 period between 2006 and 2012, a 1 : 3 matched-case-control study was conducted among children <5 years of age and resident in Puglia.

A* case* was defined as a child born between January 2006 and June 2012, hospitalized (when aged at least 6 months) for IPD or pneumococcal pneumonia (PP) between June 2006 and December 2012. The study included 28 pediatric wards of all hospitals in the region (admitting about 60,000 children aged ≤60 months per year). Hospitalization records were extracted from the regional discharge registry. IPD was defined as ICD9-CM (International Classification of Diseases, Ninth Revision, Clinical Modification) code 320.1 (pneumococcal meningitis) or 038.2 (pneumococcal septicemia) or as each of the following codes: 320.8 (the other specified meningitis), 790.7 (bacteremia), or 038.9 (unspecified septicemia) if associated with 041.2 (bacterial infection in conditions classified elsewhere and of unspecified site—pneumococcus). Pneumonia with diagnosed* S. pneumoniae *infection was defined as ICD9-CM code 481 (*Streptococcus pneumoniae *pneumonia) [17]. Records were selected if these codes were either in the main or in the secondary discharge diagnosis.

A* control* was defined as a presumed healthy child, matched by gender, age (±1 month), and municipality, retrieved from the general population registry, and checked for previous history of hospital admission due to pneumococcal diseases (exclusion criteria).

Information on vaccination status against pneumococcus was retrieved from the regional immunization registry. According to the vaccine received, a case was defined as “fully vaccinated” if the child had been vaccinated with ≥3 doses of PCV7/PCV13 at least one month before the date of hospitalization; a fully vaccinated control was a child who had received ≥3 PCV7/PCV13 doses. A case was defined as “incompletely vaccinated” if the child had received <3 doses of PCV7/PCV13 at least one month before the hospitalization; an incompletely vaccinated control was a child who had received <3 doses of vaccine.

IPD and PP cases were described by age, sex, and vaccination status. Odds Ratios (ORs) with 95% confidence intervals (95% CIs) and the Exact McNemar Test were computed by using Stata MP 12 for Mac OS tool for matched studies. Post hoc power analysis was conducted after the study had been completed and used the obtained sample size and effect size to determine what the power was in the study, assuming the effect size in the sample is equal to the effect size in the population [18]. Overall and outcome specific VE was calculated as 1 − OR with 95% CIs.

The study was conducted in accordance with The Guidelines for Good Clinical Practice and the ethical principles that originate in the Declaration of Helsinki and within the Italian law. The protocol was approved by the Institutional Review Board at the Regional Observatory for Epidemiology.

### 2.2. Impact of the Vaccination Programme

In order to assess the impact of the vaccination programme in Italy, the disease burden on the population of children <5 years of age was estimated, choosing as indicators the hospitalization rates for pneumococcal diseases before (average annual hospital admission rates between 2001 and 2005) and after (annual rates between 2006 and 2011) the introduction of the pneumococcal vaccination.

Episodes of pneumococcal diseases were extracted from the national hospital discharge registry [19] and specific hospitalization rates were calculated for the following outcomes:

- *IPD*, ICD9-CM codes as defined above;
- *PP*, ICD9-CM code as defined above;
- *All-cause pneumonia*, defined as ICD9-CM codes 480.xx–486.xx without mention of a diagnosis of IPD as defined above;
- *Unspecified AOM*, defined as ICD9-CM codes 382.xx—*suppurative and unspecified otitis media *[17].

These ICD9-CM codes were scanned across discharge diagnoses in each child record for any mention of these diseases. The hospitalization rates in the pre- and postvaccination period and the hospitalization risk ratios (HRRs) with 95% CIs were computed using outcome specific Poisson regression models [17]. National data and data from the first eight Italian regions that introduced PCVs in 2006 were computed. Analysis was performed by using STATA MP 12 for Mac OS.

## 3. Results

### 3.1. PCVs Programme Effectiveness

A total of 39 cases were recruited, nine of IPD (four males, seven aged <24 months) and 30 of PP (15 males, seven aged <24 months) (Table 1). Each case was matched to three controls. Both cases and controls were citizens of Italy.

Twenty-seven/39 (69.23%, eight IPD and 19 PP) cases had been fully vaccinated with ≥3 doses of PCV7/PCV13 compared to 107/117 (91.4%) vaccinated controls (26 among those matched to IPD and 81 among those matched to PP). The overall PCVs programme effectiveness was 75% (95% CI: 61%–84%). It was 69% (95% CI: 30%–88%) and 77% (95% CI: 61%–87%) against IPD and PP, respectively (Table 2).

### 3.2. Impact of the Vaccination Programme

Reduction in hospitalization rates for IPD was observed either at a national level or in the first eight regions that had introduced PCVs since 2006, with HRR of 0.66 (95% CI: 0.52–0.84) and 0.51 (95% CI: 0.32–0.81), respectively. A decrease was also recorded for AOM, with HRR of 0.61 (95% CI: 0.58–0.65) and 0.63 (95% CI: 0.57–0.70), respectively. In contrast, the hospitalization rates for PP increased either in Italy (HRR: 1.4, 95% CI: 1.25–1.57) or in the eight regions (HRR: 1.57, 95% CI: 1.26–1.967) (Table 3).

## 4. Discussion

Our findings provide further evidence of pneumococcal conjugate vaccination effectiveness against IPD in children aged <5 years, although data of 69% (95% CI: 30%–88%) was slightly lower than what had been estimated by the same authors in a previous study [17] and from other similar studies [9, 20]. In a postlicensure assessment of serotype-specific vaccine effectiveness from Andrews et al. in England, Wales, and Northern Ireland, PCV13 effectiveness after two doses before the age of 12 months or one dose from 12 months was 75% (95% CI: 58%–84%) [21]. It is important to notice that, compared to other studies estimating effectiveness against vaccine-type IPD, this case-control design evaluated hospital discharge records where serotype information is not reported.

To our knowledge this is the first study in Italy assessing the effectiveness of PCVs in preventing severe pneumococcal pneumonia in children. Our finding of 77% (95% CI: 61%–87%) corroborates the reduction in hospitalization rates for PP observed in Puglia after the introduction of the vaccine (HRR: 0.43, 95% CI: 0.21–0.90) [17]. A systematic review of PCV dosing from Whitney et al. summarized studies of pneumonia endpoints documenting drops in disease rates or reported cases following PCV introduction, on a range of schedules [22].

A limitation of this case-control study could be seen in the relatively small number of cases recruited. This was predictable given the high vaccination coverage reported in our territory; however the post hoc estimation of the power of the study was fairly high (84.6%) (Table 3).

The PCVs programme has showed a significant impact on invasive disease and AOM in children aged <5 years both at a national level and in those regions with a longer vaccination history, with nearly a 40% reduction of hospitalizations for both outcomes. A Danish nationwide population-based cohort study observed a 71% reduction (95% CI: 62%–79%) in IPD incidence in children aged <2 years [23]. In a study from Norway, a reduced risk of maternal report of AOM was identified among children who had received three or more PCV7 immunizations by 12 months of age (RR: 0.86; 95% CI: 0.81–0.91) and by 18 months (RR: 0.92, 95% CI: 0.90–0.94), respectively, when compared with nonimmunized children [24, 25].

In contrast, hospitalization rates for PP have increased in the most recent years. A main aspect influencing this data is that the number of hospitalizations for pneumonia has showed a peak in all age groups during and after the flu pandemic in 2009 [10].

Moreover, surveillance of pneumococcal diseases presents a number of challenges because of differences in surveillance systems and reporting practices among Italian regions, therefore producing false trends [11]. In addition, underascertainment remains considerable for the scarce attitude to investigate cases using adequate laboratory tests, as the large number of the discharge records coded as all-cause pneumonia in this study has showed. In general, the use of hospital discharge diagnoses might either underestimate or overestimate the number of pneumococcal outcomes as it relies on the review of the discharge forms (where laboratory confirmation is not reported) and not on the review of the medical records of the children hospitalized for diseases caused by or related to* S. pneumoniae*.

In conclusion, the PCVs programme has confirmed its effectiveness against the most severe cases of* S. pneumoniae* diseases and its substantial impact on nasopharyngeal carriage in children aged <5 years. Our findings indicate the importance of maintaining high routine immunization coverage, especially in a mature programme, such as in Italy, in which indirect (herd) effects help enhance protection.

## Figures and Tables

**Table 1:** Cases of IPD and pneumococcal pneumonia recruited among children hospitalized between June 2006 and December 2012 in the Puglia region, by gender, age, and year of admission.

	IPD		Pneumococcal pneumonia		Total cases	
	(*N* = 9)		(*N* = 30)		(*N* = 39)	
	*N*	%	*N*	%	*N*	%
Gender						
Male	4	*44.44 *	15	*50 *	19	*48.72 *
Female	5	*55.56 *	15	*50 *	20	*51.28 *
Age						
<24 months	7	*77.78 *	7	*23.33 *	14	*35.90 *
24–<60 months	2	*22.22 *	23	*76.67 *	25	*64.10 *
Year of admission						
2006	—	—	1	*3.32 *	1	*2.56 *
2007	1	*11.11 *	—	—	1	*2.56 *
2008	1	*11.11 *	2	*6.67 *	3	*7.69 *
2009	1	*11.11 *	2	*6.67 *	3	*7.69 *
2010	1	*11.11 *	11	*36.67 *	12	*30.78 *
2011	2	*22.22 *	8	*26.67 *	10	*25.64 *
2012	3	*33.34 *	6	*20.00 *	9	*23.08 *

**Table 2:** Cases of IPD and pneumococcal pneumonia and matched controls. PCVs effectiveness (95% CIs) in Puglia, 2006–2012.

Vaccination status	IPD cases (*N* = 9)		Matched controls (*N* = 27)		OR (95% CI)	*p* ^*∗*^	VE (95% CI)
	*N*	%	*N*	%			
Fully vaccinated^#^	8	*88.89 *	26	*96.30 *	0.31 (0.12–0.7)	0.003	69% (30%–88%)
Incompletely vaccinated^##^	1	*11.11 *	0	*0 *	n.c.^*∗∗*^	0.31	
Not vaccinated	0	*0 *	1	*3.70 *	Ref.	Ref.	

Vaccination status	Pneumococcal pneumonia cases		Matched controls		OR (95% CI)	*p* ^*∗*^	VE (95% CI)
	(*N* = 30)		(*N* = 90)				
	*N*	%	*N*	%			

Fully vaccinated^#^	19	*63.33 *	81	*90.00 *	0.23 (0.13–0.39)	<0.001	77% (61%–87%)
Incompletely vaccinated^##^	11	*36.67 *	7	*7.78 *	1.57 (0.56–4.78)	>0.05	
Not vaccinated	0	*0 *	2	*2.22 *	Ref.	Ref.	

Vaccination status	Total cases (*N* = 39)		Matched controls (*N* = 117)		OR (95% CI)	*p* ^*∗*^	VE (95% CI)
	*N*	*% *	*N*	*% *			

Fully vaccinated^#^	27	*69.23 *	107	*91.46 *	0.25 (0.16–0.39)^*∗∗∗*^	<0.001	75% (61%–84%)
Incompletely vaccinated^##^	12	*30.77 *	7	*5.98 *	1.71 (0.62–5.13)	>0.05	
Not vaccinated	0	*0 *	3	*2.56 *	Ref.	Ref.	

^#^ *Fully vaccinated cases*: children vaccinated with ≥3 doses of PCV7/PCV13 at least one month before the date of hospitalization; *fully vaccinated control*: presumed healthy children vaccinated with ≥3 doses.

^##^ *Incompletely vaccinated cases*: children vaccinated with <3 doses of PCV7/PCV13 at least one month before the date of hospitalization; *incompletely vaccinated control*: presumed healthy children vaccinated with <3 doses.

^*∗*^Exact McNemar significance probability. ^*∗∗*^n.c.: not calculable. ^*∗∗∗*^ *Power of estimation: 84.6%*.

**Table 3:** Hospitalization rates (per 100,000) and HRRs (95% CIs) for IPD, *Streptococcus pneumoniae* pneumonia, all-cause pneumonia, and AOM in the pre- and postvaccination period in Italy and in the first eight regions that introduced PCVs in 2006.

Italy	2001–2005		2006–2011		HRR (95% CI)	
	*N*	Rate per 100,000	*N*	Rate per 100,000		
IPD	163	6.14	112	4.04	0.66	(0.52–0.84)
Pneumococcal pneumonia	488	18.37	715	25.74	1.4	(1.25–1.57)
All-cause pneumonia	21,406	805.76	22,354	804.90	0.99	(0.98–1.02)
AOM	2,658	100.07	1.703	61.33	0.61	(0.58–0.65)

Eight regions which introduced PCV in 2006	2001–2005		2006–2011		HRR (95% CI)	
	*N*	Rate per 100,000	*N*	Rate per 100,000		

IPD	53	5.49	28	2.90	0.51	(0.32–0.81)
Pneumococcal pneumonia	127	13.23	205	21.40	1.57	(1.26–1.96)
All-cause pneumonia	7,287	760.09	7,430	775.03	0.99	(0.95–1.02)
AOM	870	90.79	565	58.94	0.63	(0.57–0.70)
